# Selective Decline of Synaptic Protein Levels in the Frontal Cortex of Female Mice Deficient in the Extracellular Metalloproteinase ADAMTS1

**DOI:** 10.1371/journal.pone.0047226

**Published:** 2012-10-11

**Authors:** Matthew D. Howell, Antoni X. Torres-Collado, M. Luisa Iruela-Arispe, Paul E. Gottschall

**Affiliations:** 1 University of Arkansas for Medical Sciences, Department of Pharmacology and Toxicology, Little Rock, Arkansas, United States of America; 2 University of California, Los Angeles, Department of Molecular, Cell, and Developmental Biology, Los Angeles, California, United States of America; University of Toronto, Canada

## Abstract

The chondroitin sulfate-bearing proteoglycans, also known as lecticans, are a major component of the extracellular matrix (ECM) in the central nervous system and regulate neural plasticity. Growing evidence indicates that endogenous, extracellular metalloproteinases that cleave lecticans mediate neural plasticity by altering the structure of ECM aggregates. The bulk of this *in vivo* data examined the matrix metalloproteinases, but another metalloproteinase family that cleaves lecticans, a disintegrin and metalloproteinase with thrombospondin motifs (ADAMTS), modulates structural plasticity *in vitro*, although few *in vivo* studies have tested this concept. Thus, the purpose of this study was to examine the neurological phenotype of a mouse deficient in ADAMTS1. *Adamts1* mRNA was absent in the ADAMTS1 null mouse frontal cortex, but there was no change in the abundance or proteolytic processing of the prominent lecticans brevican and versican V2. However, there was a marked increase in the perinatal lectican neurocan in juvenile ADAMTS1 null female frontal cortex. More prominently, there were declines in synaptic protein levels in the ADAMTS1 null female, but not male, frontal cortex beginning at postnatal day 28. These synaptic marker declines did not affect learning or memory in the adult female ADAMTS1 null mice when tested with the radial-arm water maze. These results indicate that *in vivo Adamts1* knockout leads to sexual dimorphism in frontal cortex synaptic protein levels. Since changes in lectican abundance and proteolytic processing did not accompany the synaptic protein declines, ADAMTS1 may play a nonproteolytic role in regulating neural plasticity.

## Introduction

The extracellular matrix (ECM) in the central nervous system (CNS) surrounds perikarya and synapses, and functionally it is important in the regulation of neural plasticity [1]. The ECM aggregate in the CNS contains hyaluronan, chondroitin sulfate (CS)-bearing proteoglycans (PGs) termed lecticans, link protein, and the glycoprotein tenascin [2]. The lecticans–aggrecan, brevican, neurocan, and versican–are substituted with a variable number of glycosaminoglycan CS chains. Perineuronal nets (PNNs) are a specialized form of the ECM that surrounds a minor subset of mainly inhibitory, parvalbumin-positive interneurons in the CNS [3]. Lecticans in PNNs and the neuropil are the key components of brain ECM that contribute to structural and functional plasticity, including the closure of critical periods early in development [4] and the stabilization of fear memories [5]. Further, lecticans are a major inhibitory component of the glial scar that develops after CNS injury, and they cause impaired axonal sprouting and regeneration and poor functional recovery [6]. While treatment with the bacterial enzyme chondroitinase ABC, which cleaves CS from lecticans, increased neurite outgrowth and improved functional recovery after CNS injury [7], [8], lectican core proteins maintained at least partial neurite outgrowth inhibition in the absence of CS [9], [10].

Two families of endogenous, extracellular metalloproteinases cleave lecticans: matrix metalloproteinases (MMPs) and a disintegrin and metalloproteinase with thrombospondin motifs (ADAMTSs). The ADAMTS enzymes are zinc-dependent, glutamyl endopeptidases with physiological roles in ECM remodeling, angiogenesis, and cell motility [11], [12]. These metalloproteinases are synthesized as zymogens before proteolytic activation and secretion into the ECM. The family member ADAMTS1 plays a crucial role in ovulation [13], [14], and knockout of the gene results in reduced fertility in female mice [15], growth retardation, renal lesions that mimic nephropathy, and adrenal gland and bladder dysfunction [16], [17], [18]. However, the neurological phenotype of this mouse has not been characterized. Indeed, the physiological expression and role of ADAMTS1 in the CNS is poorly defined.

Evidence indicates that the ECM modulates neural plasticity *in vitro*. When the ECM surrounding synapses was enzymatically removed from a culture of mature primary neurons, trafficking of glutamate AMPA receptors from extrasynaptic to synaptic sites increased [19], a phenomenon which may play an important role in long-term potentiation (LTP). Since ADAMTS1, as well as ADAMTS4, 5, 9, and 15 cleave lecticans and are secreted by neurons, astrocytes, and microglia [20], they may be involved in neural plasticity through disruption of the ECM aggregate. Primary neurons transfected with ADAMTS4 cDNA or proteolytically inactive ADAMTS4 cDNA exhibited increased neurite outgrowth [21]. This finding suggests that the ADAMTSs may play a multimodal role in neuroplasticity mediated by cleavage of lecticans or through direct interaction with neurons, possibly via their thrombospondin motif. Nothing is known about the developmental role of the ADAMTSs in neural plasticity. The purpose of this study was to examine the neurological phenotype of a mouse lacking *Adamts1* (ADAMTS1 null mouse), including expression of *Adamts* genes with lecticanase activity, abundance and proteolytic processing of lecticans, and synaptic protein levels as a relative measure of synapse abundance. We found diminished levels of synaptic proteins in the frontal cortex of female, but not male, ADAMTS1 null mice with no change in proteolytic processing of lecticans, which may suggest a non-proteolytic role for ADAMTS1 in neural plasticity.

## Materials and Methods

### Ethics Statement

All animal protocols were approved by the Institutional Animal Care and Use Committee at the University of Arkansas for Medical Sciences (Animal Use Protocol #3106), and every effort was made to the limit the number of mice used for these experiments.

### ADAMTS1 Null Mouse

The ADAMTS1 null mouse contained a global knockout of *Adamts1* [18], and a colony of mice was maintained in our vivarium. The line was rederived by Charles River Laboratories (Wilmington, MA), and heterozygote pairs were used to maintain the line and produce experimental animals. Weaned mice were housed 3–5 per cage under standard laboratory conditions with access to food and water *ad libitum*. Male and female mice were used at the following ages (P refers to postnatal days): P8 (n = 14 per genotype), P28 (n = 8–9 per genotype), P90 (n = 10 per genotype), P130–160 (n = 12 per genotype), and P180 (n = 8 per genotype).

### Radial-Arm Water Maze (RAWM)

A modified version of the RAWM [22] was used to examine spatial learning and memory in ADAMTS1 null mice. P130–160 wildtype and knockout mice (5 males and 7 females per genotype) were randomly divided into two cohorts and housed separately during testing; the experimenter was blinded to the genotype of each mouse during testing. The RAWM apparatus and experiment design were similar to that previously described [23]. The water in the pool was 20°C during all experimentation. Each mouse was assigned a goal arm that remained the same for all trials. The visual cues were black or white geometric shapes as well as the experimenter, and so the experimenter remained at the same place throughout all trials. On day one the mice were trained on the RAWM. Each cohort was divided into two equal groups. For the first trial, the first mouse was released from a random start arm and had 60 seconds to find a hidden platform (all black terra cotta pot 2 mm below the water surface) in its goal arm. An error was considered an entry into an arm without the platform or 15 seconds without entering an arm or remaining in an incorrect arm. If the mouse did not locate the platform after 60 seconds it was gently guided to it and the mouse remained on the platform for 10 seconds. After each trial the mouse was dried with a washcloth and placed under a heat lamp. This protocol was repeated until each mouse in the first group was tested for six trials. The second group was then tested while the first group rested. The first group was then tested for three additional trials followed by the second group. The next day (day two), the mice were tested for the ability to recall the platform location based upon the spatial cues. Testing was similar to day one except each group was tested for six trials, followed by a rest, followed by three trials, followed by a rest, and followed by three additional trials. To examine long-term memory recall, mice were tested four weeks after day two (day thirty) in an identical manner to day two. The average number of errors for each block of three trials (block 1 was trials one through three, block 2 was trials four through six, et cetera) was calculated and used for analysis. Previous testing with this mouse model indicated that the mice learned the location of the platform too quickly when a visible platform was utilized. Thus, to slow learning, the number of trials each mouse was tested was decreased and a hidden platform was used for all trials.

### Tissue Preparation

Mice were euthanized with an overdose of carbon dioxide and the brain was removed from the skull. Brain regions including frontal cortex, hippocampus and cerebellum were dissected and stored at −80°C until further processing. P180 mice were transcardially perfused with phosphate buffered saline (PBS) followed by 4% (w/v) paraformaldehyde. The brains were removed, post-fixed in 4% paraformaldehyde overnight at 4°C, and then stored in PBS with 0.04% sodium azide at 4°C until processed for immunohistochemistry.

Protein was extracted from brain regions using radio immunoprecipitation assay (RIPA) buffer (50 mM Tris-HCl, 150 mM NaCl, 2 mM EDTA, 1% sodium deoxycholate, 0.1% sodium dodecyl sulfate [SDS], 1% Triton-X-100) with a 1∶100 dilution of protease inhibitor cocktail III (Calbiochem, La Jolla, CA). The tissue was homogenized with 10 volumes of RIPA buffer in a 2 ml glass homogenization tube with a Teflon-coated homogenizer (20 up and down motions). The homogenate was transferred to a 1.5 ml microfuge tube and then centrifuged at 21,100 x *g* for 15 minutes at 4°C. The supernatant was removed from the pellet and the bicinchoninic acid (BCA) protein assay (Thermo-Pierce, Rockford, IL) was used to determine the protein concentration. Extracts were stored in aliquots at −80°C.

### Immunoblotting

Sample extracts with equal protein amounts (15 µg for brevican and fragments and 30 µg for versican and fragments, neurocan, and TIMP-3) were combined with 2x Laemlli sample buffer, heated at 95°C for 4 minutes, loaded onto Tris-Glycine 6% or 4–20% gradient SDS-PAGE gels (Invitrogen, Carlsbad, CA), and electrophoresed. Proteins were then electrophoretically transferred onto polyvinylidine difluoride membrane (PVDF, Immobilon, Millipore, Billerica, MA) and probed for various antigens. The blots were washed in buffer B (PBS, 0.05% Tween-20, pH 7.4) for five minutes and then blocked in buffer B containing 5% dry nonfat milk (SaCo, Middleton, WI) for one hour. All membranes were incubated in primary antibody overnight at 4°C. The primary antibodies and dilutions were: mouse anti-brevican (1∶1000; BD Biosciences, San Jose, CA); rabbit anti-EAMESE (1∶1000) [24]; rabbit anti-SAHPSA (1∶1000) [25]; mouse anti-versican V2 (12C5, 1∶1000; DSHB, Iowa City, IA); rabbit anti-NIVNSE (1∶500) [23]; rabbit anti-PLPDSR (1∶500; Gottschall Lab); mouse anti-neurocan (1F6, 1∶1000; DSHB); rabbit anti-TIMP-3 (1∶500; Cell Signaling); rabbit anti-GAPDH (1∶5000; Cell Signaling, Beverly, MA). The blots were then incubated with goat anti-mouse IgG or goat anti-rabbit IgG conjugated to horse radish peroxidase (HRP) (Millipore, Temecula, CA) diluted 1∶20,000. Antigens were visualized with the Immobilon Western Chemiluminescent HRP Substrate (Millipore) and the blots were exposed using the Kodak 4000MM imager (Carestream Health, Rochester, NY) or to autoradiography film (Denville Scientific, Metuchen, NJ).

### Quantification of Immunoblots

The Kodak Molecular Imaging Software (Carestream Health) was used to measure the mean intensity of each antigen band as well as the background for each blot. For the blots with P8 and P28 samples, the film was scanned at 400 dpi with the Epson Perfection V700 Photo scanner (Epson, Long Beach, CA), converted to 8-bit grayscale, the background subtracted, the color inverted, and the mean intensity for each band measured with ImageJ software. For each method the background was subtracted from each sample and this value was then divided by the mean intensity for GAPDH for the same blot to obtain normalized mean intensity. For each antigen the wildtype and ADAMTS1 null normalized mean intensity values were divided by the average mean intensity for all wildtype samples to obtain percent of wildtype.

### Enzyme-Linked Immunosorbent Assay (ELISA)

Sandwich ELISAs for the pre-synaptic membrane protein synaptosome-associated protein 25 kD (SNAP-25), the vesicular glycoprotein synaptophysin, and the postsynaptic scaffolding protein postsynaptic density 95 kD (PSD-95) were performed as described [26]. The capture antibodies and dilutions were: mouse anti-SNAP-25 (1∶200; Millipore), mouse anti-synaptophysin (1∶200; Millipore) and mouse anti-PSD-95 (1∶250; Neuromab, Davis, CA). The detection antibodies and dilutions were: rabbit anti-SNAP-25 (1∶1000; Sigma-Aldrich, Saint Louis, MO), rabbit anti-synaptophysin (1∶1000; Santa Cruz Biotechnology, Santa Cruz, CA) and rabbit anti-PSD-95 (4 µg/mL; Millipore). The secondary antibody was goat-anti rabbit IgG conjugated to HRP (Millipore) diluted 1∶1000 (synaptophysin and PSD-95) or 1∶5000 (SNAP-25). A standard curve was constructed from the supernatant of a RIPA-extracted whole mouse brain with a selected dilution (near the beginning of saturation) designated as 100 units of binding activity [26]. The units for each synaptic protein obtained with ELISA were divided by the protein concentration for each sample to obtain units synaptic protein per µg protein.

### Immunohistochemistry, Image Acquisition, and Analysis

Each fixed P180 brain was sequentially cryoprotected in 15% and 30% sucrose overnight at 4°C, mounted on a cryostat chuck with Optimal Cutting Temperature (OCT) medium (Sakura Finetek USA, Torrance, CA), equilibrated at −22°C, and sectioned at 30 µm with a Microm HM505E cryostat. Sections were stored free floating in PBS until used for immunohistochemistry. Two sections per brain including frontal cortex were washed in PBS 3 times for 5 minutes each, blocked and permeabilized in 10% normal goat serum, 3% 1 M lysine, and 0.3% Triton-X-100 for 1 hour and then incubated overnight at 4°C with biotinylated *Wisteria floribunda* agglutinin (WFA; Vector Laboratories, Burlingame, CA) diluted 1∶1000. The sections were then washed in PBS and incubated in streptavidin-Alexa Fluor 488 (Invitrogen) diluted 1∶1000. Sections were mounted on Superfrost/Plus glass slides (Fisher Scientific, Pittsburg, PA) with Vectashield Mounting Medium with DAPI (Vector Laboratories).

Images were acquired with a Zeiss AxioImager.Z1 microscope using a 20x PlanApo lens and AxioVision software (Carl Zeiss Microscopy and Imaging, Jena, Germany) using the same gain and intensity. TIFF files were exported for analysis with ImageJ software. The polygon tool was used to draw a boundary around each PNN to determine the mean intensity and the area. The experimenter was blinded to the genotype during image acquisition and analysis.

### RNA Isolation and Quantitative Real-Time Polymerase Chain Reaction (RT-PCR)

Total RNA was isolated from P8, P28, and P130–160 frontal cortex using the NucleoSpin RNA II kit (Clontech, Mountain View, CA). Total RNA concentration was determined using the NanoDrop ND-1000 Spectrophotometer (Thermo Scientific, Waltham, MA) and 1 µg of RNA was reverse transcribed to cDNA using the High Capacity cDNA Reverse Transcription Kit (Applied Biosystems, Foster City, CA). Quantitative RT-PCR was performed on the ABI7900HT Fast Real-Time PCR System using TaqMan Gene Expression Master Mix and TaqMan Gene Expression Assays (Applied Biosystems) for the following genes: *Adamts1* (Assay ID Mm00477355_m1), *Adamts4* (Assay ID Mm00556068_m1), *Adamts5* (Assay ID Mm00478620), *Adamts9* (Assay ID Mm00614433_m1), *Adamts15* (Assay ID Mm01176187_m1), and *Gapdh* (Assay ID Mm03302249_g1). Each sample was assayed in triplicate and the average logarithmic Ct value was converted to the linear form using the conversion 2^−Ct^ [27]. For each sample linear Ct values for the *Adamts* genes were divided by the linear Ct *Gapdh* value to obtain relative transcript abundance (normalized to *Gapdh).*

### Statistics

All statistical analyses were performed with GraphPad Prism 5 (GraphPad Software, La Jolla, CA). Data from the RAWM was analyzed with repeated measures two-way analysis of variance (ANOVA) with Bonferroni pairwise comparison. Relative transcript abundance data was compared with two-way ANOVA (genotype×age) with Bonferonni pairwise comparison. ELISA and densitometry values were analyzed with Student’s *t* test (two-tailed) at each age for each sex. Values for PNN mean intensity/area and area were analyzed with the Mann-Whitney U test. For all tests p<0.05 was considered a significant difference. All data is shown as mean ± standard error of the mean (S.E.M.).

## Results

### Relative Transcript Abundance of Adamts Genes during Development

**Figure 1:**
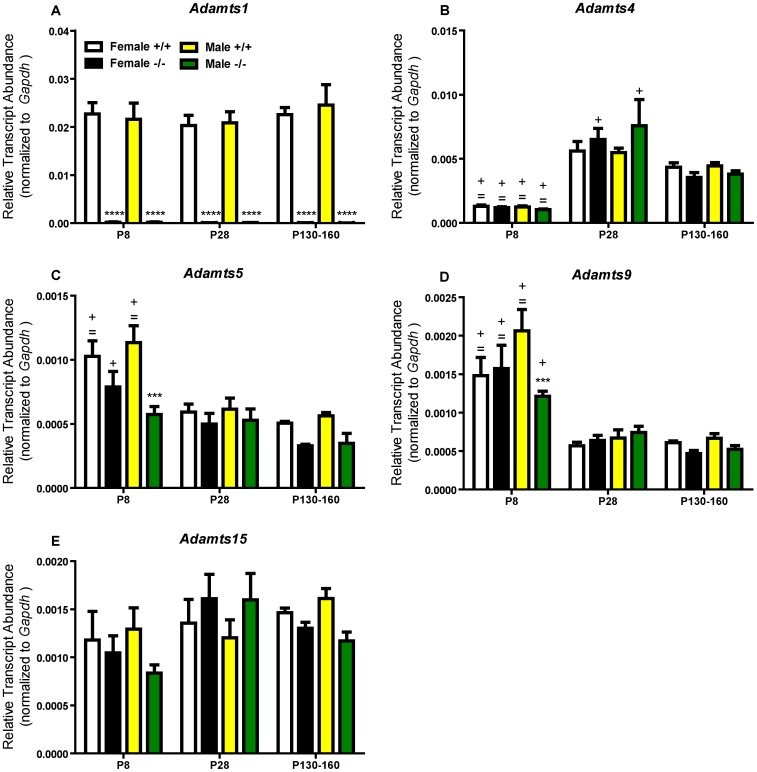
Relative transcript abundance of *Adamts* genes in the ADAMTS1 null and wildtype frontal cortex. Relative transcript abundance for (A) *Adamts1*, (B) *Adamts4* (C) *Adamts5*, (D) *Adamts9*, and (E) *Adamts15* at P8, P28, and P130–160 in ADAMTS1 null (−/−) and wildtype (+/+) frontal cortex as measured by quantitative RT-PCR using TaqMan assays. For each sample (n = 4–7 at each age, sex, and genotype), the logarithmic Ct value was converted to the linear form with 2^−Ct^ and then divided by the linear *Gapdh* value to obtain relative transcript abundance normalized to *Gapdh*. For each gene, ADAMTS1 null and wildtype values (separated by sex) were compared with two-way ANOVA (genotype×age) followed by Bonferroni pairwise comparison; ***p<0.001 and ****p<0.0001 indicate a difference from the same sex and age wildtype mice; = p<0.05 indicates a significant difference from the same sex and genotype P28 average; +p<0.05 indicates a significant difference from the same sex and genotype P130–160 average.

Transcripts of various *Adamts* genes that code for enzymes that cleave lecticans were measured in ADAMTS1 null and wildtype frontal cortex at three ages to determine developmental expression levels of *Adamts* genes and whether there were compensatory changes in other *Adamts* mRNA due to lack of *Adamts1*. Quantitative RT-PCR revealed a low basal level of *Adamts1* mRNA in male and female ADAMTS1 null frontal cortex at P8, P28, and P130–160 (Figure 1A). Interestingly, *Adamts1* does not appear to be differentially expressed during development because the relative transcript abundance was similar at all three developmental time points in male and female wildtype frontal cortex. *Adamts5* and *Adamts9* transcript abundance was significantly reduced in the male (but not female) P8 ADAMTS1 null compared to wildtype frontal cortex with no difference at P28 or P130–160 (Figure 1C and 1D). Temporally, *Adamts4* was more highly expressed at P28 and P130–160 compared to P8 for all mice (Figure 1B), *Adamts5* and *Adamts9* transcript abundances were generally highest at P8 (Figure 1C and 1D), and *Adamts15* was expressed relatively consistently at all ages (Figure 1E). There was no compensatory upregulation of any *Adamts* gene observed in the ADAMTS1 null frontal cortex.

### Lectican Abundance and Processing in the ADAMTS1 Null Frontal Cortex

**Figure 2:**
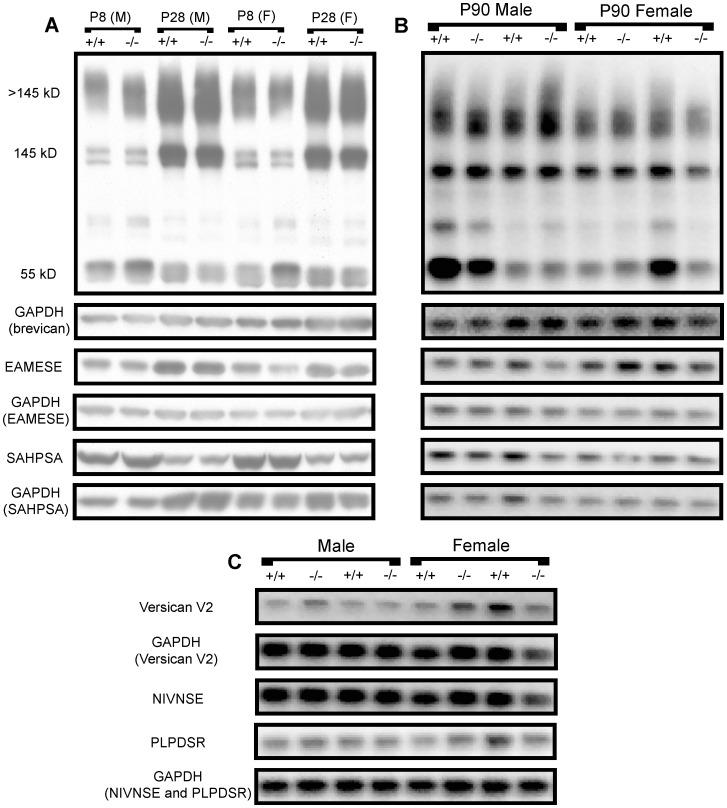
Brevican abundance and proteolytic processing in ADAMTS1 null and wildtype frontal cortex extracts. (A) Representative immunoblots from female (F) and male (M) P8 and P28 ADAMTS1 null (−/−) and wildtype (+/+) frontal cortex protein extracts which show brevican (>145 kD, 145 kD, and 55 kD), the 55 kD ADAMTS-derived brevican fragment (EAMESE), the 53 kD MMP-derived brevican fragment (SAHPSA), and GAPDH from the corresponding immunoblots. (B) Representative immunoblots from P90 ADAMTS1 null and wildtype frontal cortex protein extracts with the same bands as in (A). (C) Representative immunoblots for P90 ADAMTS1 null and wildtype frontal cortex protein extracts which show intact versican V2 (245 kD), the ADAMTS-derived 60 kD versican fragment (NIVNSE), the MMP-derived 43 kD versican fragment (PLPDSR), and GAPDH from the corresponding immunoblots.

**Figure 3:**
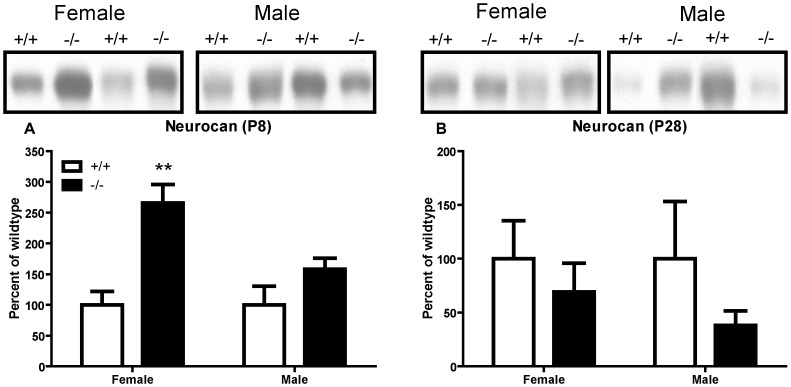
Neurocan abundance in P8 and P28 ADAMTS1 null and wildtype frontal cortex extracts. Representative immunoblots and densitometry for female and male (A) P8 and (B) P28 ADAMTS1 null (−/−) and wildtype (+/+) protein extracts. For each sample (n = 4–5 for each age, genotype and sex), the mean intensity for neurocan was expressed as a percent of wildtype (no GAPDH normalization since these were 6% gels; equal protein amounts were added to the gels). The values for genotype, separated by age and sex, were compared with two-tailed Student’s *t* test; **p<0.01 indicates a difference from the wildtype same sex, same age mouse group.

We hypothesized that absence of *Adamts1* in the ADAMTS1 null frontal cortex would increase the abundance of intact lecticans and decrease their proteolytic processing. Brevican, although most abundant in the adult ECM [28], was easily measured in perinatal brain region protein extracts [29]. Therefore, the abundance and ADAMTS-dependent proteolytic processing of brevican were measured in male and female ADAMTS1 null and wildtype P8, P28, and P90 frontal cortex extracts (Figure 2A and 2B). There was no significant difference in the abundance of brevican bearing CS (>145 kD), brevican core protein without CS (145 kD), the 55 kD proteolytic fragment of brevican (from MMP and ADAMTS cleavage), the ADAMTS-derived 55 kD fragment (EAMESE), or the MMP-derived 53 kD fragment (SAHPSA) between male or female ADAMTS1 null and wildtype frontal cortex extracts (Figure 2A and 2B and Figure S1). In P90 mice, there was also no significant difference in the abundance of the adult-restricted lectican versican V2 holoprotein (245 kD),the ADAMTS-derived 60 kD fragment (NIVNSE), or the MMP-derived 43 kD fragment (PLPDSR) in either female or male ADAMTS1 null and wildtype frontal cortex extracts (Figure 2C and Figure S2). Neurocan, a lectican found most prominently in the perinatal ECM [28], was measured in P8 and P28 frontal cortex extracts; there was a marked 260% increase in intact neurocan (∼270 kD) in P8 female, but not male, ADAMTS1 null compared to wildtype frontal cortex extracts (Figure 3A). Further, there was no difference in neurocan in P28 male or female ADAMTS1 null or wildtype frontal cortex extracts (Figure 3B). Additionally, the protein level of the endogenous ADAMTS inhibitor, tissue inhibitor of metalloproteinase-3 (TIMP-3), was no different between ADAMTS1 null and wildtype frontal cortex extracts at any age (data not shown).

### PNNs in ADAMTS1 Null Frontal Cortex

While there was no global change in lectican abundance or processing in adult ADAMTS1 null frontal cortex, changes may occur more discretely in PNNs. Thus, PNNs were analyzed in horizontal brain sections stained with WFA, a lectin which binds N-acetylgalactosamine in CS. There was no significant difference in the staining intensity per area or area for male or female ADAMTS1 null or wildtype PNNs (data not shown), which indicates that PNNs in ADAMTS1 null and wildtype frontal cortex were no different in either size or lectican content. Overall, ADAMTS1 knockout resulted in a marked increase in neurocan in P8 female (but not male) frontal cortex extract, but no measurable changes in the lectican composition of the adult ECM.

### Synaptic Protein Levels in ADAMTS1 Null Frontal Cortex

**Figure 4:**
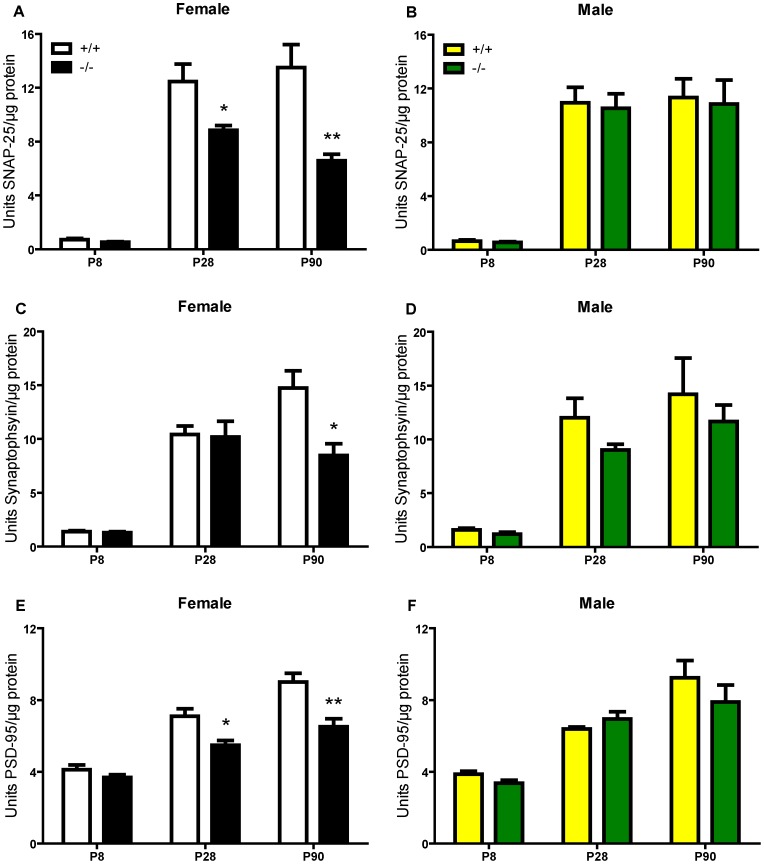
Synaptic protein levels in P8, P28, and P90 ADAMTS1 null and wildtype frontal cortex extracts. The synaptic proteins (A and B) SNAP-25, (C and D) synaptophysin, and (E and F) PSD-95 were measured in frontal cortex protein extracts from female and male ADAMTS1 null (−/−) and wildtype (+/+) mice at P8, P28, and P90 (n = 4–7 per age, sex, and genotype). The values for units synaptic protein/µg protein were compared with a two-tailed Student’s *t* test between genotypes for each sex at each age; *p<0.05 and **p<0.01 indicates a difference from the wildtype same sex, same age mouse group.

Since lecticans mediate neural plasticity, changes in lectican abundance and processing and/or direct action of ADAMTS1 on neurons, especially early in development, could influence synapse formation. Our lab developed several ELISAs to measure synaptic proteins [26], and we used these as a relative measure of synapse abundance in ADAMTS1 null and wildtype frontal cortex extracts during development. At P8, there was no significant difference in the levels of the presynaptic proteins SNAP-25 or synaptophysin and the postsynaptic protein PSD-95 between ADAMTS1 null and wildtype frontal cortex extracts (Figure 4). However, beginning at P28, there was a significant decline in SNAP-25 and PSD-95 and, at P90, marked declines in all three synaptic markers in female ADAMTS1 null frontal cortex (Figure 4A, 4C, and 4E). There was no significant difference in synaptic protein levels in male ADAMTS1 null compared to wildtype frontal cortex extracts at any age (Figure 4B, 4D, and 4F). Further, this effect was limited to frontal cortex since there were no differences in synaptic marker levels in P90 male or female ADAMTS1 null or wildtype hippocampal or cerebellar extracts (Tables S1 and S2), although there was a trend for a decrease in the synaptophysin level in male ADAMTS1 null hippocampus that nearly reached statistical significance.

### Learning and Memory in the ADAMTS1 Null Mouse

**Figure 5:**
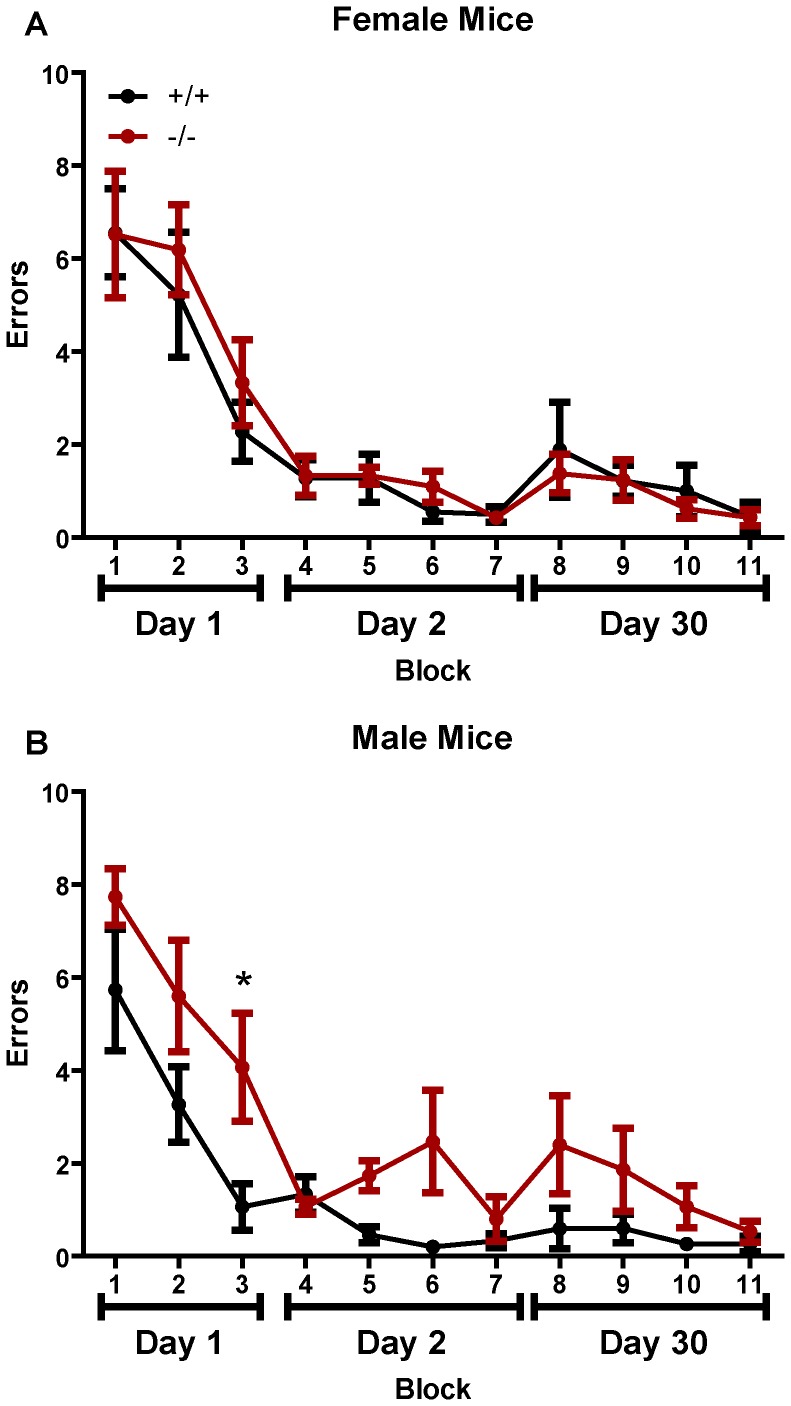
Spatial learning and memory in ADAMTS1 null and wildtype mice. Spatial learning and memory was assessed in P130–160 male and female ADAMTS1 null (−/−) and wildtype (+/+) mice (n = 5–7 per sex and genotype) with the RAWM. Each block represents three trials and thus mice were tested for nine trials on day 1, twelve trials on day 2, and twelve trials on day 30. The average number of errors made in locating the platform is shown for (A) female and (B) male mice. The data were analyzed with a two-way repeated measures ANOVA (see text for details) followed by Bonferroni pairwise comparison; *p<0.05 indicates a significant difference between the ADAMTS1 null and wildtype mice at that time point.

The marked declines in synaptic proteins in the female ADAMTS1 null frontal cortex may indicate impairments in learning and memory. Therefore, we examined spatial learning and memory in male and female ADAMTS1 null and wildtype mice using the RAWM. Although spatially-mediated learning and memory is thought to primarily involve the hippocampus, we also examined long-term recall which has some input from cortex. Regardless of sex and genotype, all mice initially made approximately an average of 7 errors during the first block (trials 1 through 3) of testing (Figure 5). Performance improved for all female mice over the first day, and the mice ended at around an average of 3 errors for block 3 (Figure 5A). However, male ADAMTS1 null mice performed worse than the wildtypes, and by block 3 (end of day 1), made significantly more errors compared to wildtype littermates (Figure 5B). On day two all female mice performed similarly and averaged less than 1 error by the end of testing (Figure 5A). Male ADAMTS1 and wildtype mice began and ended with the same number of errors, although the ADAMTS1 null mice made more errors during blocks 5 and 6 (Figure 5B). When measuring long-term recall on day thirty, all mice performed similarly and ended making well less than 1 error on the final block. There was a significant genotype effect for the male mice [*F*(1,8) = 12.73, p = 0.0073], but not for female mice [*F*(1,10) = 0.8315, p = 0.7469] when analyzed with repeated-measures two-way ANOVA. Overall, the male ADAMTS1 null mice appeared to learn the location of the platform slower than the wildtype mice, but since both genotypes performed similarly by the end of testing there appears to be no memory deficit in the ADAMTS1 null mice.

## Discussion

Mounting evidence indicates that extracellular metalloproteinases contribute to the regulation of neural plasticity. For example, MMP-9 inhibition impaired spatial learning [30] and maintenance of LTP [31], while exogenous MMP-9 induced dendritic spine enlargement and caused synapse potentiation [32]. Each of these outcomes was related to proteolytic cleavage of a target substrate. Less is known about the role that a second metalloproteinase family, the ADAMTSs, may play in neural plasticity. *In vivo,* increased ADAMTS-dependent proteolytic cleavage of lecticans was associated with regenerative sprouting after entorhinal cortex lesion [24] and after kainate-induced seizure [33]. *In vitro*, ADAMTS4 mediated neurite outgrowth even in the absence of proteolytic activity [21]. In the current study, we found declines in several synaptic proteins in frontal cortex extracts of female, but not male, ADAMTS1 null compared to wildtype mice. Further, these effects did not appear to be associated with altered abundance and proteolytic processing of lectican substrates in the adult, although there was a significant increase in intact neurocan in P8 female ADAMTS1 null frontal cortex. While male ADAMTS1 null mice displayed no significant change in synaptic protein levels, there was an apparent delay in spatial learning, but otherwise there were no deficits in spatial learning and memory in the ADAMTS1 null mouse.

Quantitative RT-PCR results indicated the absence of *Adamts1* transcript in the ADAMTS1 null frontal cortex and, assuming equal affinity across various *Adamts* TaqMan assays, ADAMTS1 was the most highly expressed of all the *Adamts* genes assayed in the wildtype frontal cortex at all ages. Thus, it is possible that since ADAMTS1 is effective at cleaving all of the lectican substrates, the absence of the gene would result in marked modifications in the abundance and processing of lecticans. Interestingly, brevican and versican V2 abundance and proteolytic processing were not altered in female or male ADAMTS1 null mice at any age while neurocan, which is highly expressed during perinatal development, increased markedly in the P8 female ADAMTS1 null compared to wildtype frontal cortex extracts. While neurocan does contain a possible ADAMTS cleavage site [34], it is unknown if it is cleaved *in vivo* by ADAMTSs, although it was cleaved *in vitro* by MMP-2 [35] and with a high concentration of ADAMTS4 [36]. These observations could indicate that the other ADAMTSs with lecticanase activity compensate for the absence of ADAMTS1 in the ADAMTS1 null frontal cortex, although there was no significant increase in mRNA expression in any of these proteinases. Further, it is unknown if there is preferential cleavage of lecticans by any particular ADAMTS in the CNS based on individual cell type expression and/or location in the extracellular microenvironment. The relative abundance of the *Adamts1* transcript was highest of the *Adamts* genes assayed in this study, but this does not necessarily indicate that this gene has the highest translation into protein and thus the highest relative activity among the ADAMTS in the frontal cortex. While *Adamts5* and *Adamts9* relative transcript abundance decreased in P8 male ADAMTS null compared to wildtype frontal cortex, they were no different from wildtype at P28 and P130–160 and these changes did not appear to affect lectican processing or synaptic protein levels. Further, while there were no compensatory increases in other *Adamts* transcript abundances in ADAMTS1 null frontal cortex, there may have been compensation in appropriate lectican cleavage. ADAMTS1 knockout did not alter MMP-dependent cleavage of brevican or versican V2 since the MMP-derived fragments of these lecticans were no different between ADAMTS1 null and wildtype frontal cortex extracts.ADAMTS1 knockout also did not alter the frontal cortex protein level of the endogenous ADAMTS inhibitor TIMP-3 at any age.

The most intriguing finding in this study was the synaptic protein declines observed in female, but not male, ADAMTS1 null compared to wildtype frontal cortex extracts. The restriction of these declines to female mice could indicate they are related to and/or dependent upon estrogen. The level of estrogen influences dendritic spine density in the hippocampus; decreased spine density on CA1 pyramidal neurons accompanied decreased estrogen levels [37]. Further, estradiol treatment following ovariectomy restored dendritic spines to pre-ovariectomy levels [38], [39]. While the ADAMTS1 null female exhibits impaired fertility, namely related to impaired follicular development and ovulation [40], the estrous cycle was normal [15]. Additionally, the declines in synaptic proteins were significant by P28 in the female ADAMTS1 null frontal cortex, an age that precedes sexual maturity and thus suggests the declines are not dependent upon a lack of estrogen in the brain. While the declines were selectively observed in frontal cortex, an estrogen sensitive region of the brain [41], [42], estrogen-dependent fluctuations in dendritic spine density have not been reported in this brain region. The exact mechanism by which ADAMTS1 knockout affects only females, and specifically only frontal cortex, is not known. Perhaps the effect is mediated in part by an estrogen-dependent receptor that when combined with knockout of ADAMTS1 results in deficient synaptogenesis. While male ADAMTS1 null mice displayed no significant changes in frontal cortex synaptic protein levels there was a non-significant trend for a decline in synaptophysin in the ADAMTS1 null hippocampus as well as a delay in spatial learning in the RAWM. This delay could be mediated by a decline in synaptophysin–as a relative measure of synapse abundance–in discrete regions of the hippocampus masked by the whole tissue; however, by the end of the task male ADAMTS1 null and wildtype mice do not perform differently in the RAWM.

Compensation from the other lecticanase ADAMTS family members could explain why there was little change in lectican abundance and proteolytic processing. However, the marked decreases in synaptic proteins in the female ADAMTS1 null frontal cortex might suggest a non-proteolytic ADAMTS1 effect. Indeed, *in vitro*, neurons transfected with ADAMTS4 cDNA exhibited increased neurite outgrowth, even in the absence of proteolytic activity, through activation of ERK-1/2 [21]. This finding suggested that ADAMTS4 (and possibly the structurally similar ADAMTS1) could bind directly to a neuronal surface receptor to initiate signaling that increased neurite outgrowth. Unpublished observations from our lab also demonstrated that ADAMTS4 shifted dendritic spines *in vitro* to the more immature, filopodial shape and a more plastic state. Perhaps *in vivo* ADAMTS1 mediates physiological synapse formation and stabilization, and knockout of the gene results in deficient synapse formation. While it is not known which ADAMTS1 domain would bind to a receptor and mediate its effects, ADAMTS1 does contain three thrombospondin type 1 repeats, and thrombospondin was shown to increase neurite outgrowth *in vitro* [43]. Exogenous addition of thrombospondin-1 and -2 promoted synaptogenesis *in vitro* and knockout of both genes resulted in reduced synaptic puncta *in vivo* [44]. Although the EGF domains of thrombospondin (not present in ADAMTS1) were required for synaptogenesis in one study [45], an ECM molecule with thrombospondin type-1 repeats, heparin-binding growth-associated molecule, increased neurite outgrowth in hippocampal neurons [46]. Further, *in vitro*, thrombospondin 1 exerted an apparent synaptogenic effect through binding to neuroligin 1 [47] and so ADAMTS1 deficiency could result in decreased clustering of neuroligin 1 and thus decreased synaptogenesis. Interestingly, addition of MMP-9 to primary neuronal cultures increased the lateral mobility of the NR1 subunit of the glutamate NMDA receptor, an effect dependent upon integrin β1 signaling but not MMP-9 proteolytic activity [48]. Although ADAMTS1 and MMP-9 do not share homology in their structure, ADAMTS1 may also participate in integrin signaling. It is also possible that ADAMTS1 cleaves non-lecitcan substrates which influence synaptic changes. Indeed, ADAMTS1 cleaved thrombospondin-1 and -2 *in vitro* and in the skin epithelium [49]. While it is unknown if this proteolysis occurs in the CNS, perhaps ADAMTS1 cleaves thrombospondin to release fragments involved in synapse formation and/or stabilization, and the lack of this normal cleavage may be involved in the synaptic declines in female ADAMTS1 null frontal cortex. Future studies to identify ADAMTS1 binding proteins on the neuronal surface, and possible ADAMTS1 substrates, could shed light into a potentially unexplored area of neuroplasticity mediated directly by metalloproteinases.

This study suggests for the first time that ADAMTS1 is associated with *in vivo* neural plasticity. Although the MMPs have been implicated in changes with neuroplasticity, including structural changes with dendritic spines and functional changes in synaptic plasticity, the possible role that ADAMTSs may play has not been well examined. The novel implications that ADAMTS1–and possibly other ADAMTS family members–may play a multimodal role in plasticity opens up further areas of research that may be able to target or manipulate these proteinases in efforts to improve structural and functional recovery in CNS injury and disease.

## Supporting Information

Figure S1 **Densitometry for brevican abundance and proteolytic processing in ADAMTS1 null and wildtype frontal cortex extracts.** Densitometric analysis from the immunoblots in figure 2A and 2B is shown in the graphs separated by sex: female (A) P8, (C) P28, and (E) P90, and male (B) P8, (D) P28, and (F) P90. For each sample (n = 3–7 for each age, sex, and genotype), the mean intensity for the band of interest was divided by the GAPDH mean intensity and then expressed as a percent of the wildtype average. There were no significant differences between ADAMTS1 null and wildtype mice of the same sex at any age.
(TIF)

Figure S2 **Densitometry for versican abundance and proteolytic processing in ADAMTS1 null and wildtype frontal cortex extracts.** (A) Representative versican V2 immunoblot that shows the versican V2 antibody recognizes the intact proteoglycan at 245 kD as well as a faint band at 60 kD, which probably represents the 60 kD ADAMTS-derived N-terminal versican fragment. Densitometric analysis from the immunoblots in figure 2C are shown for (B) female and (C) male P90 ADAMTS1 null (−/−) and wildtype (+/+) frontal cortex protein extracts. For each sample (n = 3–4 for each sex and genotype), the mean intensity for the band of interest was divided by the GAPDH mean intensity and then expressed as a percent of the wildtype average. There were no significant differences between ADAMTS null and wildtype mice of the same sex at any age.
(TIF)

Table S1 **Synaptic protein levels in P90 ADAMTS1 null (−/−) and wildtype (+/+) hippocampal protein extracts.** Values represent units synaptic protein/µg total protein. Data are expressed as mean ± S.E.M. (n = 5 mice per sex and genotype).
(DOCX)

Table S2 **Synaptic protein levels in P90 ADAMTS1 null (−/−) and wildtype (+/+) cerebellar protein extracts.** Values represent units synaptic protein/µg total protein. Data are expressed as mean ± S.E.M. (n = 5 mice per sex and genotype).
(DOCX)
