# Supplementary figures and images for: Selective Decline of Synaptic Protein Levels in the Frontal Cortex of Female Mice Deficient in the Extracellular Metalloproteinase ADAMTS1
Source: PLoS One. 2012 Oct 11;7(10):e47226. doi: 10.1371/journal.pone.0047226 (PMC3469530; doi:10.1371/journal.pone.0047226)

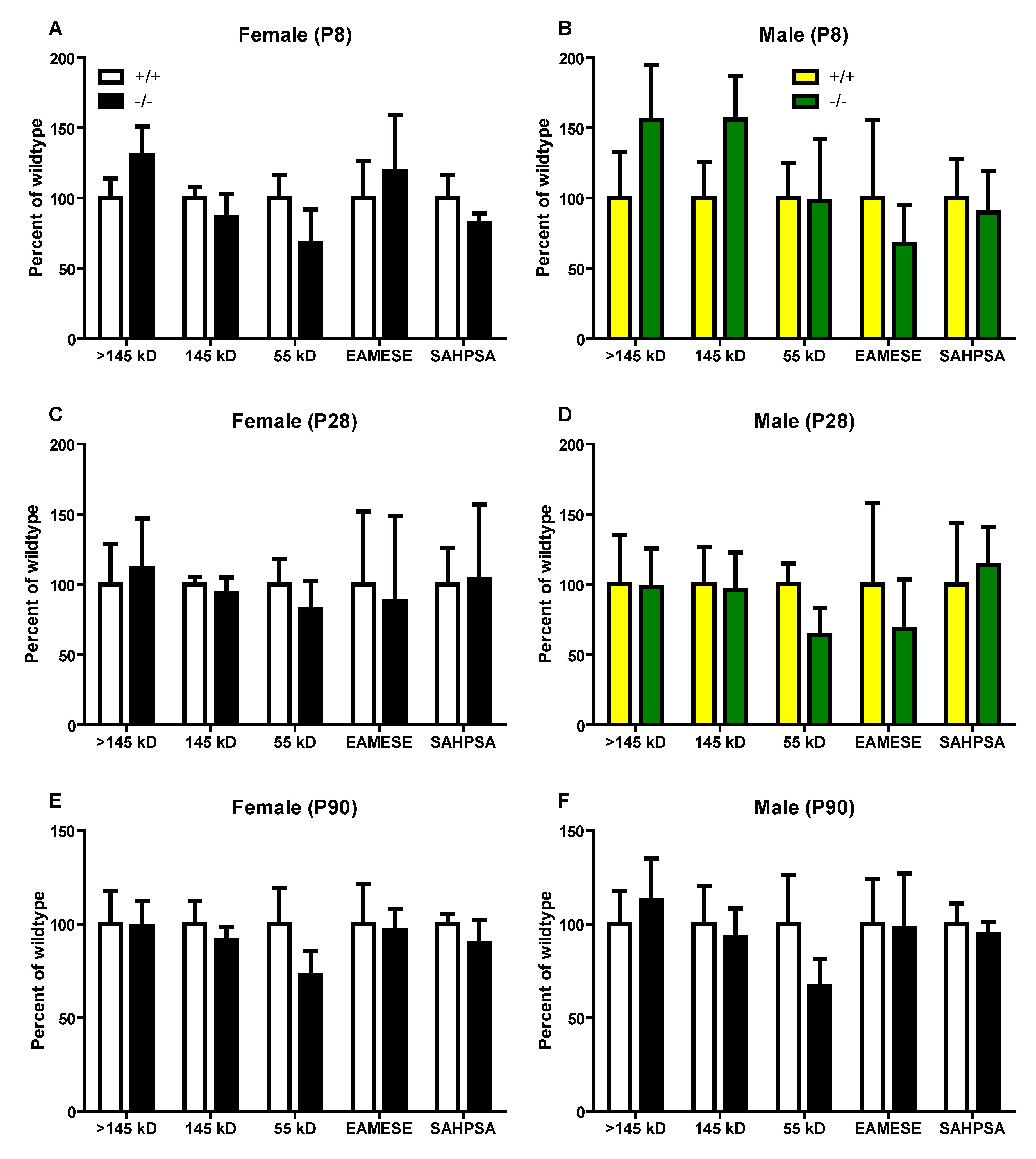

Supplement: Figure S1 — Densitometry for brevican abundance and proteolytic processing in ADAMTS1 null and wildtype frontal cortex extracts. Densitometric analysis from the immunoblots in figure 2A and 2B is shown in the graphs separated by sex: female (A) P8, (C) P28, and (E) P90, and male (B) P8, (D) P28, and (F) P90. For each sample (n = 3–7 for each age, sex, and genotype), the mean intensity for the band of interest was divided by the GAPDH mean intensity and then expressed as a percent of the wildtype average. There were no significant differences between ADAMTS1 null and wildtype mice of the same sex at any age. (TIF) [file pone.0047226.s001.tif]

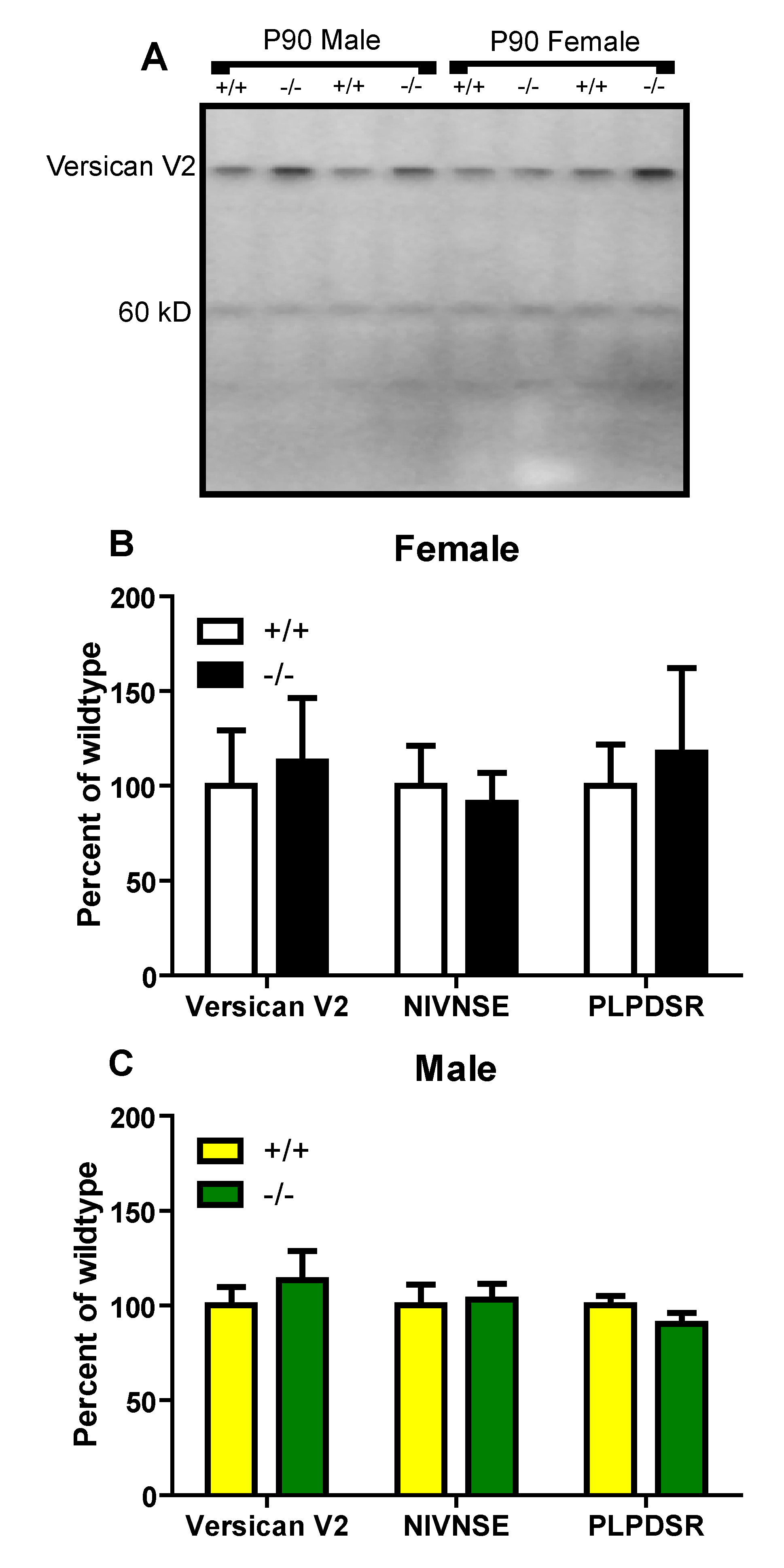

Supplement: Figure S2 — Densitometry for versican abundance and proteolytic processing in ADAMTS1 null and wildtype frontal cortex extracts. (A) Representative versican V2 immunoblot that shows the versican V2 antibody recognizes the intact proteoglycan at 245 kD as well as a faint band at 60 kD, which probably represents the 60 kD ADAMTS-derived N-terminal versican fragment. Densitometric analysis from the immunoblots in figure 2C are shown for (B) female and (C) male P90 ADAMTS1 null (−/−) and wildtype (+/+) frontal cortex protein extracts. For each sample (n = 3–4 for each sex and genotype), the mean intensity for the band of interest was divided by the GAPDH mean intensity and then expressed as a percent of the wildtype average. There were no significant differences between ADAMTS null and wildtype mice of the same sex at any age. (TIF) [file pone.0047226.s002.tif]
