# Supplementary material for: Selective Decline of Synaptic Protein Levels in the Frontal Cortex of Female Mice Deficient in the Extracellular Metalloproteinase ADAMTS1
Source: PLoS One. 2012 Oct 11;7(10):e47226. doi: 10.1371/journal.pone.0047226 (PMC3469530; doi:10.1371/journal.pone.0047226)
Supplement: Table S1 — Synaptic protein levels in P90 ADAMTS1 null (−/−) and wildtype (+/+) hippocampal protein extracts. Values represent units synaptic protein/µg total protein. Data are expressed as mean ± S.E.M. (n = 5 mice per sex and genotype). (DOCX) [file pone.0047226.s003.docx]

| **Synaptic Protein** | **Female** | | **Male** | |
| --- | --- | --- | --- | --- |
|  | **+/+** | **-/-** | **+/+** | **-/-** |
| **SNAP-25** | 7.05 ± 0.581 | 6.49 ± 0.498 | 6.28 ± 0.442 | 5.83 ± 0.596 |
| **Synaptophysin** | 11.7 ± 0.933 | 10.2 ± 0.826 | 10.5 ± 0.977 | 7.80 ± 0.848 |
| **PSD-95** | 7.72 ± 0.998 | 7.08 ± 0.271 | 6.51 ± 0.400 | 7.08 ± 0.770 |
