# Supplementary material for: Selective Decline of Synaptic Protein Levels in the Frontal Cortex of Female Mice Deficient in the Extracellular Metalloproteinase ADAMTS1
Source: PLoS One. 2012 Oct 11;7(10):e47226. doi: 10.1371/journal.pone.0047226 (PMC3469530; doi:10.1371/journal.pone.0047226)
Supplement: Table S2 — Synaptic protein levels in P90 ADAMTS1 null (−/−) and wildtype (+/+) cerebellar protein extracts. Values represent units synaptic protein/µg total protein. Data are expressed as mean ± S.E.M. (n = 5 mice per sex and genotype). (DOCX) [file pone.0047226.s004.docx]

| **Synaptic Protein** | **Female** | | **Male** | |
| --- | --- | --- | --- | --- |
|  | **+/+** | **-/-** | **+/+** | **-/-** |
| **SNAP-25** | 7.02 ± 0.986 | 9.62 ± 0.700 | 5.79 ± 0.609 | 6.10 ± 0.594 |
| **Synaptophysin** | 5.96 ± 0.141 | 7.30 ± 0.866 | 5.39 ± 0.316 | 6.62 ± 0.349 |
| **PSD-95** | 3.21 ± 0.250 | 3.01 ± 0.172 | 3.00 ± 0.153 | 2.93 ± 0.170 |
